# Polymorphisms of the *FCN2* Gene 3’UTR Region and Their Clinical Associations in Preterm Newborns

**DOI:** 10.3389/fimmu.2021.741140

**Published:** 2021-10-28

**Authors:** Anna S. Świerzko, Dariusz Jarych, Gabriela Gajek, Karolina Chojnacka, Paulina Kobiela, Maja Kufelnicka-Babout, Mateusz Michalski, Katarzyna Sobczuk, Agnieszka Szala-Poździej, Misao Matsushita, Jan Mazela, Iwona Domżalska-Popadiuk, David C. Kilpatrick, Jarosław Kalinka, Hideharu Sekine, Maciej Cedzyński

**Affiliations:** ^1^ Laboratory of Immunobiology of Infections, Institute of Medical Biology, Polish Academy of Sciences, Łódź, Poland; ^2^ Department of Newborns’ Infectious Diseases, Poznań University of Medical Sciences, Poznań, Poland; ^3^ Department of Neonatology, Medical University of Gdańsk, Gdańsk, Poland; ^4^ Department of Perinatology, First Chair of Gynecology and Obstetrics, Medical University of Łódź, Łódź, Poland; ^5^ Department of Applied Biochemistry, Tokai University, Hiratsuka, Japan; ^6^ Scottish National Blood Transfusion Service, National Science Laboratory, Edinburgh, Scotland, United Kingdom; ^7^ Department of Immunology, Fukushima Medical University, Fukushima, Japan

**Keywords:** 3’UTR, ficolin-2, *FCN2*, newborn, prematurity

## Abstract

Ficolin-2 is regarded as an important innate immunity factor endowed with both lectin (carbohydrate recognition) qualities and ability to induce complement activation. The aim of this study was to investigate the association of the *FCN2* 3’-untranslated region (3’UTR) polymorphisms with ficolin-2 expression and perinatal complications in preterm neonates. The sequencing analysis allowed us to identify six 3’UTR polymorphisms with minor allele frequency (MAF) >1%: rs4521835, rs73664188, rs11103564, rs11103565, rs6537958 and rs6537959. Except for rs4521835, all adhered to Hardy-Weinberg expectations. Moreover, rs6537958 and rs6537959 were shown to be in perfect linkage disequilibrium (LD) with nine other genetic polymorphisms: rs7040372, rs7046516, rs747422, rs7847431, rs6537957, rs6537960, rs6537962, rs11462298 and rs7860507 together stretched on a distance of 1242 bp and very high LD with rs11103565. The 3’UTR region was shown to bind nuclear extract proteins. The polymorphisms at rs4521835 and rs73664188 were found to influence serum ficolin-2 concentration significantly. All polymorphisms identified create (together with exon 8 polymorphism, rs7851696) two haplotype blocks. Among 49 diplotypes (D1-D49) created from rs7851696 (G>T), rs4521835 (T>G), rs73664188 (T>C), rs11103564 (T>C), rs11103565 (G>A) and rs6537959 (T>A), twenty two occurred with frequency >1%. Two diplotypes: D13 (GTTTGT/GGTCGT) and D10 (GTTTGT/GGTCGA), were significantly more frequent among preterm neonates with early onset of infection and pneumonia, compared with newborns with no infectious complications (OR 2.69 and 2.81, respectively; both p<0.05). The minor (C) allele at rs73664188 was associated with an increased risk of very low (≤1500 g) birthweight (OR=1.95, p=0.042) but was associated with the opposite effect at rs11103564 (OR=0.11, p=0.005).

## Introduction

Ficolin-2 (L-ficolin) is a serum pattern-recognition molecule, which is able to accelerate clearance of microorganisms directly as an opsonin and indirectly by complement activation *via* the lectin pathway.

Ficolin-2 has been shown to recognise numerous clinically relevant isolates like enteroaggregative *Escherichia coli, Aspergillus fumigatus, Mycobacterium tuberculosis, Streptococcus pneumoniae, Staphylococcus aureus* and *Trypanosma cruzi* (1–5). Lipoteichoic acids, peptidoglycan, 1,3-β-glucans or mycobacterial Ag85 complex have been identified as ficolin-2 microbial targets (6–8). Moreover, ficolin-2 was shown to interact with endogenous factors like elastin, complement CR1 receptor, C-reactive protein (CRP) and long pentraxin 3 (9–12) and may be involved in the clearance of late apoptotic/necrotic cells (13). The CRP-ficolin-2 complex, formed under inflammatory conditions, enables a cross-talk between lectin and classical pathways of complement activation that results in enhancement of serum antimicrobial activity (11).

The *FCN2* gene contains 8 exons and has been localised to chromosome 9 (9q34) (14). Exon 1 encodes the 5’UTR, the signal peptide (S) and the N-terminal fragment of the mature protein; exons 2 and 3 encode the collagen-like domain (C and C2), exon 4 encodes a linker peptide (L), exons 5-7 encode part of the fibrinogen like (FBG) domain (F1-F3), and exon 8 encodes the rest of the FBG and the 3’UTR (14). Three splicing variants have been identified: the first variant lacks the second exon sequence and has an additional 76 bp sequence extending from 3’UTR; the second variant is generated by the insertion of the fifth intron and also has an additional 3’UTR sequence of 1060 bp; and the third variant is generated by the insertion of both third and fifth introns (14).

The concentration of ficolin-2 in serum is known to depend on polymorphisms in both *FCN2* promoter and structural regions. Hummelshoj et al. (15) and Kilpatrick et al. (16) demonstrated significant associations of ficolin-2 levels with promoter polymorphisms at positions -986 (rs3124952, A>G), -602 (rs3124953, G>A), -64 (rs7865453, A>C) and -4 (rs17514136, A>G). Two exon 8 single nucleotide polymorphisms (SNPs) (rs7851696 and rs17549193, at positions +6424 (G>T) and +6359 (C>T), respectively) were also shown to affect ficolin-2 concentration in serum/plasma, due to strong linkage disequilibria (LD) with rs7865453 (-64) and rs17514136 (-4), respectively (17, 18). Those two exon 8 SNPs influence the structure of the fibrinogen-like domain and the affinity of ficolin-2 to its ligands (12, 15). However, marked variations among individuals carrying the same polymorphic variants were reported and genotype-independent factors seem also to influence ficolin-2 serum concentration (16). For example, cord blood serum ficolin-2 level was shown to be associated with gestational age and birthweight (19). Furthermore, Troldborg et al. (20) found significantly lower serum ficolin-2 in female compared with male adult blood donors. In patients suffering from malaria, markedly higher serum ficolin-2 levels were also observed in the acute phase of disease than after treatment (21). Huge intra-genotype differences in ficolin-2 concentrations may lead to the difficulties in data interpretation and identification of ficolin-2 genotype/phenotype disease associations - especially in studies with a restricted number of samples. Therefore, genotyping alone cannot be used to predict serum concentration. However, an influence of other, still undefined polymorphisms on ficolin-2 serum level cannot be excluded.

93% of functional polymorphisms in the genome-wide association study (GWAS) catalogue are located in non-coding regions (22). Such polymorphisms can affect gene splicing, binding of transcription factors and the interaction between transcript and microRNA – posttranscriptional regulators of gene expression. Generally, 11% of human reference SNP are located in the 3’UTR and they are gaining growing attention (23). They were shown for example to have an impact on the susceptibility to bacterial or viral infections (24, 25), periodontitis (26), sepsis and risk of development of multiorgan dysfunction syndrome (MODS) in severe traumatic patients (27). Moreover, they may be associated with cancer (28–30) and pulmonary hypertension (31).

Previously, we have suggested that low ficolin-2 concentration in cord serum may contribute to the adverse consequences of prematurity (19). Since the marked intra-genotype variations in ficolin-2 levels cannot be fully explained with known promoter/exon 8 polymorphisms, we aimed to search for SNPs within *FCN2* 3’UTR and verify their biological significance using samples from neonates born preterm as a clinical model. In contrast to *FCN2* gene 5’UTR and exon 8 SNPs, knowledge about 3’UTR variability and clinical associations is very limited. To our knowledge, this is the first report concerning the *FCN2* 3’UTR genotype-phenotype relationship.

## Materials and Methods

### Subjects

Cord blood samples from 504 Polish preterm neonates including 106 extremely/early preterm (born at gestational age <33 weeks, according to the classification of The World Health Organisation, WHO) and 398 moderate/late preterm (gestational age 33-37 weeks) (**Table 1**) were obtained from the Department of Newborns’ Infectious Diseases (University of Medical Sciences, Poznań, Poland), Department of Neonatology (Medical University of Gdańsk, Poland) and Department of Perinatology (Medical University of Łódź, Poland). 326 babies came from singleton pregnancies, 172 from 97 twin pregnancies (in 22, material from only one sibling was collected) and 6 from 2 triple pregnancies. The study was approved by the corresponding local ethics committees (Bioethics Committee of The Karol Marcinkowski Poznań University of Medical Sciences, Independent Bioethics Committee for Scientific Research at Medical University of Gdańsk, Bioethics Committee of The Medical University of Łódź). Written informed parental consent was obtained. This work conforms to the provisions of the Declaration of Helsinki. Isolated serum (blood taken into tubes with clot activator) was kept at -80°C. DNA (blood taken into tubes with sodium citrate) was isolated using GeneMATRIX Quick Blood Purifaction Kit (EURx Ltd. Gdańsk, Poland), according to the manufacturer’s protocol.

**Table 1:** Basic clinical characteristics of the study group.

Parameter	All samples	Neonates		
		Extremely/early preterm	Moderate/late preterm	Statistical significance (extremely/early *vs.* moderate/late preterm)
**n**	504	106	398	–
**Gestational age**				p<0.0001
Median	35	31	35	
Mean	34	30.3	35	
Range	24-36	24-32	33-36	
**Birthweight**				
Mean	2296	1608	2476	p<0.0001
Median	2330	1615	2480	
Range	565-4630	565-3400	970-4630	
**Female/Male**	0.9	1.1	0.86	p=0.27
**Early onset of infection (%)**	72 (14.7%)	38 (36%)	34 (8.5%)	p<0.0001
**Late onset of infection (%)**	35 (6.9%)	16 (15%)	19 (4.8%)	p=0.0007
**Pneumonia**	39 (7.7%)	27 (25%)	12 (3%)	p<0.0001
**pPROM^1^ (%)**	142 (28%)	36 (34%)	106 (26.6%)	p=0.14
**RDS^2^ (%)**	107 (21%)	60 (56.6%)	47 (11.8%)	p<0.0001
**Ficolin-2^3^ (ng/ml)**				
Median	1829	1593	1909	p=0.024
Mean	1981	1705	2040	
Range	153-5644	237-4426	153-5644	

^1^preterm prelabor rupture of membranes; ^2^respiratory distress syndrome; ^3^concentration in cord serum.

### DNA Sequencing of the *FCN2* 3’UTR Region

The *FCN2* 3’UTR region was generally amplified in three separate PCRs: reaction A (amplified region on chromosome 9: 134 887 339-134 888 460), reaction B (amplified region on chromosome 9: 134 888 251-134 890 157) and reaction C (amplified region on chromosome 9: 134 889 426-134 890 521). Alternatively, in the case of problems with sequencing of the product of reaction B, the additional reaction D (amplified region on chromosome 9: 134 887 339-134 890 506) was performed. PCR conditions, as well as sequences of primers and expected PCR products lengths are listed in **Table 2**. The PCR mixtures (in a total volume of 20 µl) consisted of 50 ng of genomic DNA, 1× DreamTaq polymerase reaction buffer (includes 20 mM MgCl_2_) (Life Technologies, Carlsbad, CA, USA), 1 U DreamTaq polymerase (Life Technologies, USA), 1 mM of each deoxynucleotide, and 0.5 µM of each primer. Reactions were performed using a Veriti™ 96-Well Thermal Cycler (Thermo Fisher Scientific, Waltham, MA, USA). PCR amplicons were analyzed using horizontal 2% agarose gel electrophoresis in a 1 × TAE buffer. The 100 bp Plus DNA size marker (Thermo Fisher Scientific) was used to normalize the size of each PCR product. The PCR products were purified using the EPPiC Fast mixture (A&A Biotechnology, Gdańsk, Poland), according to the manufacturer’s protocol. For sequencing of PCR products, the BrilliantDye™ Terminator (v.3.1) Cycle Sequencing kit (Life Technologies) was used, according to the manufacturer’s protocol. For both A and C reactions – primers A_ F, A_R, C_F and C_R, were used (**Table 2**), respectively whereas for sequencing of the PCR products of B and D reactions, the forward primer GCCTGACCAGGCTTTTAGAG or reverse primer AGGTGCACACACACACACAC were employed. For the analysis of following SNP: rs4521835, rs73664188 and rs11103564 SNP, the reaction E (amplified region on chromosome 9: 134 887 339-134 888 155) was performed. The sequencing was successful in 97% at rs11103564 and 99% at rs4521835 and rs73664188. PCR products were purified with the use of BigDye XTerminator™ Purification Kit (Thermo Fisher Scientific), according to the manufacturer’s protocol and then sequenced using the 3500xl Genetic Analyzer (Applied Biosystems, San Diego, CA, USA). Sequencing results were analyzed using Chromas software version 2.4.1 (Technelysium, Brisbane, Australia).

**Table 2:** Primers and PCR conditions used for the *FCN2* gene 3’UTR sequencing.

Reaction	Primers	PCR condition	PCR product size
**A**	A_F: ATGGCATCAACTGGAAGTCG	96°C, 3 min; 38 x: (96°C, 45 s; 58°C, 20 s; 72°C, 60 s); 72°C, 7 min	1122 bp
	A_R : GGACCCTTCTGGATCCTCTC		
**B**	B_F: CCCTCTCCTCTCACCACGTC	96°C, 3 min; 61°C, 45 s; 72°C, 2 min; 38x: (96°C, 45 s; 59°C,30 s; 72°C, 2 min); 72°C, 7 min	1907 bp
	B_R: TTCTTTACCGCATCCTGACC		
**C**	C_F: GGTTTGTTGGGATAAGAAAATG	96°C, 3 min; 38x: (96°C, 45 s; 58°C, 20 s; 72°C, 60 s); 72°C, 7 min	1096 bp
	C_R: TTTGGGGCGAGTTCATAAAG		
**D**	D_F: ATGGCATCAACTGGAAGTCG	96°C, 2 min; 35x: (96°C, 60 s; 62°C, 30 s; 72°C, 4 min); 72°C, 10 min	3258 bp
	D_R: TTCACAGCTTTGGGGAAAAT		
**E**	A_F: ATGGCATCAACTGGAAGTCG	94°C, 5 min, 35x (94°C, 15 s; 56°C, 20 s; 72°C, 45 s); 72°C, 3 min	817 bp
	E_R: GGCTTTCTGATTCAATCTGC		
**F**	F_F : GCTTTTAGAGGCTCCGCAC	96°C, 5 min, 40x (96°C, 15 s 62°C, 20 s; 72°C, 45 s); 72°C, 5 min	1325bp
	F_R : TCCTACAGAGCCCTTCCGA		

### Allelic Discrimination Using TaqMan Probes

Three SNPs were analyzed using TaqMan^®^ Assays purchased from Thermo Fisher Scientific: rs6537958 (Assay ID: C_29220561_10), rs6537959 (Assay ID: C_29220562_20) and rs11103565 (Assay ID: C_31552275_10). For all three SNPs, the PCR mixture consisted of 10 µl of 2 × TaqMan™ Genotyping Master Mix (Thermo Fisher Scientific), 1 µl of appropriate 20X TaqMan^®^ Assay, and 10 ng of genomic DNA. The PCR mixture was filled up with a distilled, DNase and RNase free water (Gibco, Waltham, MA, USA) to the final volume of 20 µl. The real-time PCR was performed using the 7900HT Fast Real-Time PCR System (Applied Biosystems) with 96-Well Block Module using standard mode thermal cycling setting under the following conditions: an initial denaturation step at 95°C for 10 min, followed by 40 cycles of denaturation (95°C, 15 s), single annealing and extension step (60°C for 1 minute). Fluorescence signal detection for both dyes was performed after each cycle. For each plate, a no template control (NTC) was included. Allelic discrimination analysis was performed using a SDS 2.3 software (Applied Biosystems).

### Determination of the *FCN2* Gene Promoter and Exon 8 Polymorphisms

Promoter polymorphisms at positions -986 (rs3124952, A>G) and -602 (rs3124953, G>A) were investigated according to the procedures described by Metzger et al. (32) while those at positions -64 (rs7865453, A>C) and -4 (rs17514136, A>G) as well as exon 8 SNPs (+6359, rs17549193, C>T; +6424, rs7851696, G>T) were determined as described by Szala et al. (33), with minor modifications.

### Determination of Ficolin-2 Concentration in Cord Sera

Ficolin-2 in 395 cord blood serum samples was determined in TRIFMA as described by Świerzko et al. (34). Briefly, anti-ficolin-2 mAb (ABS 005-16, BioPorto Diagnostics, Hellerup, Denmark) were employed for 384 HB Optiplate coating, whereas biotinylated mAb (GN4, Hycult Biotech, Uden, The Netherlands) and Eu^3+^-labelled streptavidin (Perkin Elmer, Boston, MA, USA) were used for protein detection. The inter-assay and intra-assay coefficients of variation (%CV) were 18% and 13% for serum with ficolin-2 concentration of 3500 ng/ml, respectively whereas for serum with ficolin-2 concentration of 4900 ng/ml they equalled 10% and 7%, respectively.

### Electrophoretic Mobility Shift Assay

Electrophoretic Mobility Shift Assay was performed using LightShift^™^ Chemiluminescent EMSA Kit (Thermo Fisher Scientific), according to the manufacturer’s protocol. A 1325 bp DNA PCR product was obtained using F_F and F_R primers (**Table 2**). The PCR mixtures (in a total volume of 50 µl) consisted of 20 ng of genomic DNA, 1× DreamTaq polymerase reaction buffer (includes 20 mM MgCl_2_) (Life Technologies), 1 U DreamTaq polymerase (Life Technologies), 800 µM of each deoxynucleotide, and 0.4 µM of each primer. PCR product was labelled with biotin at 3’ end of the probe using the Pierce™ Biotin 3’ End DNA Labeling Kit (Thermo Fisher Scientific), according to the manufacturer’s protocol. Nuclear extract was prepared from human hepatocyte HepG2 cell line using a Nuclear Extract kit (Thermo Fisher Scientific). Four fmol of biotinylated PCR product was incubated (20 min, RT) with or without 3 µl of nuclear extract in the binding buffer supplemented with glycerol (2.5%), MgCl_2_ (5 mM), Poly (dI·dC) 50 ng/µl, NP-40 (0.05%), 50 mM KCl and 10 mM EDTA. For inhibition experiments, four fmol of biotinylated PCR product was incubated (20 min, RT) with or without 3 µl of nuclear extract, as well as with or without inhibitor (0.4 pmol of unlabelled PCR product). After electrophoresis in native 6% polyacrylamide gel in TBE buffer, DNA was transferred to Amersham Hybond-N+ nylon membrane (Cytiva, Marlborough, MA, USA) and crosslinked at 120 mJ/cm^2^ using a commercial UV-light crosslinking instrument (auto-crosslinking function). The biotin-labelled DNA was detected by chemiluminescence by placing a membrane in a film cassette and exposure membrane to X-ray for 1 minute.

### Statistics

The Hardy-Weinberg equilibrium (HWE) exact test was used for the analysis of the genotype distribution consistency for all polymorphisms. Linkage disequilibrium (LD) and haplotype block analysis were performed by Haploview 4.2 software (http://www.broad.mit.edu/mpg/haploview/). LD analysis was performed for each pair of polymorphisms using D’ and r^2^, indicating the amount of LD between two genetic loci. A value of 1 suggests no random association of alleles at different loci. Haplotype block identification was performed based on Four Gamete Rule. The PHASE software (http://stephenslab.uchicago.edu/phase/download.html, version 2.1.1.) was used for diplotype reconstruction from genotype data. *In silico* analysis was performed with SNPinfo (FuncPred) (https://snpinfo.niehs.nih.gov) and RegulomeDB (https://www.regulomedb.org) databases. The statistical significance of the genetic impact on serum levels was tested with Mann-Whitney *U* test, Kruskal-Wallis ANOVA test and Spearman Rank correlation. The frequencies of genotypes were compared by two-sided Fischer’s exact test (or χ^2^ when appropriate). The GraphPad Prism 6 software (https://www.graphpad.com) was used for those calculations. P values <0.05 were considered statistically significant. Odds ratio was calculated using online MedCalc software (https://www.medcalc.org).

## Results

### Identification of Polymorphisms in 3’UTR Region of *FCN2* Gene and Their Frequencies in Preterm Neonates

To identify the polymorphic sites in *FCN2* 3’UTR region, in the preliminary experiment the 3183 bp fragment in 75 consecutive DNA samples was analyzed *via* Sanger sequencing. Initial screening allowed us to identify fifteen polymorphic sites with MAF>0.1 (**Table 3**).

**Table 3:** Polymorphisms identified in the *FCN2* gene 3’UTR region.

	Polymorphism	HGVS^*^ variant description	Consequence type	Position on chromosome 9
1	rs4521835	c.*45T>G	3’UTR variant	134 887 460
		c.*45T>C		
		c.*45T>A		
2	rs73664188	c.*346T>C	Regulatory region variant	134 887 761
3	rs11103564	c.*614T>C	Regulatory region variant	134 888 029
4	rs11103565	c.*853G>A	Regulatory region variant	134 888 268
5	rs7040372	c.*963C>T	Regulatory region variant	134 888 378
6	rs7046516	c.*1014A>G	Regulatory region variant	134 888 429
7	rs7847422	c.*1309A>G	Regulatory region variant	134 888 724
8	rs7847431	c.*1326A>C	Regulatory region variant	134 888 741
9	rs6537957	c.*1496T>C	Regulatory region variant	134 888 911
10	rs6537958	c.*1562C>T	Regulatory region variant	134 888 977
11	rs6537959	c*1631T>A	Regulatory region variant	134 889 046
12	rs6537960	c.*1652C>T	Regulatory region variant	134 889 067
13	rs6537962	c*1674T>C	Regulatory region variant	134 889 089
14	rs11462298	c.*2009_*2010insA	Intergenic variant	134 889 424-
				134 889 425
15	rs7860507	c.*2205G>A	Intergenic variant	134 889 620

*HGVS, Human Genome Variants Society.

Next, the frequency of those genetic variants was determined in the remaining samples of preterm neonates (**Table 4**). Rs4521835, rs73664188 and rs111103564 were investigated using Sanger sequencing, whereas rs11103565 was investigated by using the TaqMan Allelic Discrimination assay. The agreement between sequencing analysis and TaqMan probes for 89 samples was 91%. Disagreement was observed in three cases, whereas in five cases the SDS 2.3 software was not able to determine the genotype. Eleven polymorphic sites located between 134 888 378 and 134 889 620 bp analyzed in the 75 samples mentioned were shown to be in complete LD. Using the SNPinfo algorithm for the Caucasian population two tag SNPs: rs6537958 (correlated with rs6537957, rs6537960, rs7040372 and rs7847431) and rs6537959 (correlated with rs7046516, rs6537962a and rs7860507) were indicated. The frequencies of rs4521835, rs73664188, rs111103564 as well as rs6537958 and rs6537959 (representing eleven polymorphic sites) in preterm neonates are presented in **Table 4**. They did not differ from those reported for the Central European (CEU) population. Except for rs4521835, all *FCN2* polymorphisms tested adhered to the Hardy-Weinberg expectations (p>0.05) (**Table 4**).

**Table 4:** The frequencies of analyzed *FCN2* 3’UTR genetic variants in polish preterm babies.

dbSNP ID	Observed genotypes^a^ (n)			HWE p value^b^	MAF^c^	MAF^d^ in population:	
	RR	RV	VV			Total	CEU
**rs4521835**	199	200	101	0.000017	0.402	0.415	0.407
**rs73664188**	377	116	7	0.2614	0.130	0.121	0.116
**rs11103564**	187	228	74	0.8611	0.384	0.309	0.312
**rs11103565**	275	184	28	0.5217	0.246	0.185	0.182
**rs6537958**	234	216	50	0.5217	0.316	0.245	0.243
**rs6537959**	234	216	50	0.5217	0.316	0.236	0.229

a Number of detected genotypes, R, reference allele; V, variant allele; ^b^p value is consistent with Hardy-Weinberg equilibrium if p>0.001; ^c^minor allele frequency; ^d^minor allele frequency reported in dbSNP [ALFA allele frequencies in total and Central European (CEU) populations].

### Linkage Disequilibrium and Haploblock Analysis

**Figure 1:**
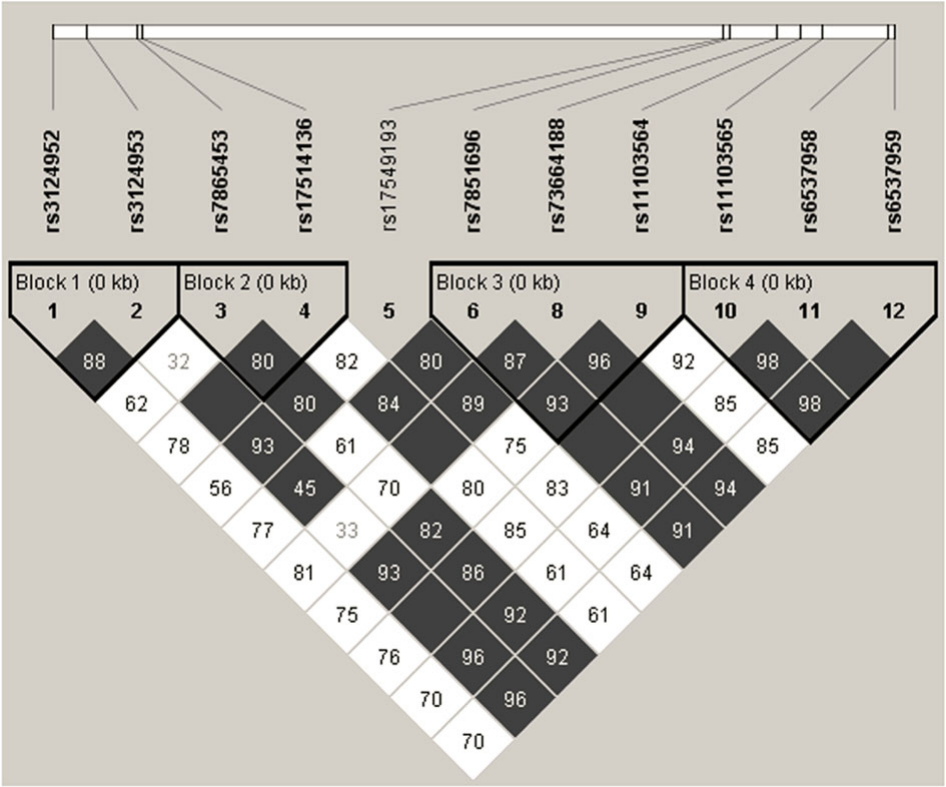
Linkage disequilibrium analysis of promoter [rs3124952 (-986); rs3124953 (-602); rs7865453 (-64); rs17514136 (-4)], exon 8 [rs17549193 (+6359); rs7851696 (+6424)] SNP and polymorphisms identified in this study (rs73664188; rs11103564; rs11103565; rs6537958; rs6537959). The numbers in the grid refer to D’ parameter (presented as percent) of the given pairs of SNPs. Empty grey squares represent perfect LD (D’=1). SNP identifiers are indicated on the abscissas. Bolded triangles shows haplotype blocks identified using Four Gamete Rule test.

Based on four *FCN2* promoter, two exon 8 and five 3’UTR polymorphisms, linkage disequilibrium analysis was performed in 504 samples (**Figure 1**). According to the r^2^ and D’ parameters, perfect linkage (r^2^ = 1, D’=1) was detected between two 3’UTR SNPs (rs6537958 and rs6537959). As a result of sequencing analysis of 75 DNA samples, a perfect LD with them was also detected for other nine 3’UTR polymorphisms (rs7040372, rs7046516, rs7847422, rs7847431, rs6537957, rs6537960, rs6537962, rs114662298 and rs7860507) and confirmed in the ensembl database (https://www.ensembl.org/Homo_sapiens/Info/Index) for the CEU population. Very high r^2^ and D’ values (0.669 and 0.98, respectively) were observed also between rs11103565 and rs6537958 as well as rs11103565 and rs6537959. Low r^2^ but high D’ values (>0.8) were observed between 3’UTR rs73664188 and promoter rs3124953 (-602), rs7865453 (-64), rs17514136 (-4), exon 8 rs7851696 (+6424) as well as 3’UTR polymorphisms (rs11103564, rs11103565, rs6537958, rs6537959 and presumably nine others mentioned above).

Since rs4521835 did not comply with Hardy-Weinberg equilibrium, it was not included in the analysis. However, in the subgroup of early preterm neonates, no deviation from HWE was found for this SNP and Haploview analysis revealed a high LD with rs73664188, rs11103564, rs11103565, rs6537958 and rs6537959. The LD plots for separated extremely/early and moderate/preterm neonates are presented in **Supplementary Materials** (**Figure S1**).

**Figure 2:**
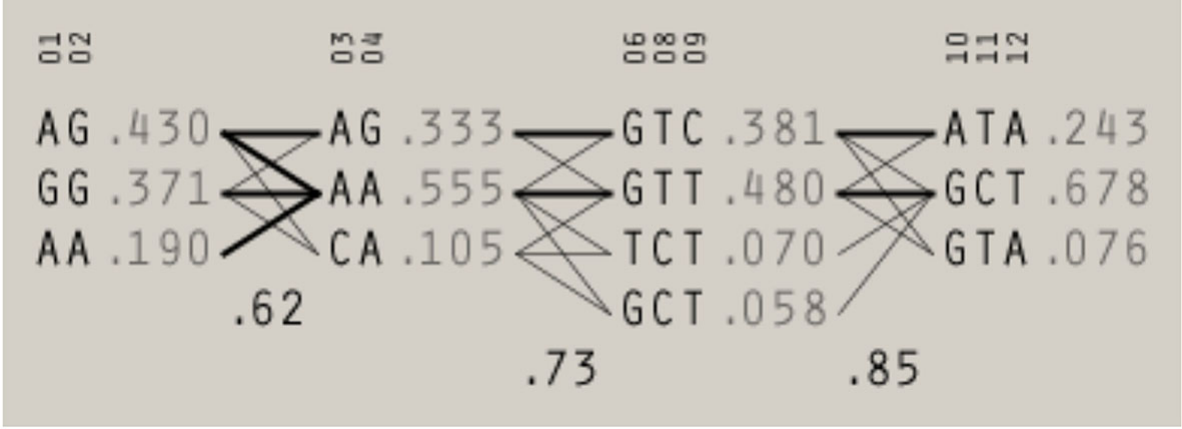
The haplotype blocks identified in preterm neonates using Four Gamete Rule test. The frequency and the level of recombination between blocks are given. 01 - rs3124952 (-986); 02 - rs3124953 (-602); 03 - rs7865453 (-64); 04 - rs17514136 (-4), 06 - rs7851696 (+6424), 08 -rs73664188; 09 - rs11103564; 10 - rs11103565; 11 - rs6537958; 12 - rs6537959. A multiallelic D’ statistic, which indicates the level of recombination between two blocks, is shown in the crossing area.

Using the Four Gamete Rule approach, four haplotype blocks were identified: block 1 and block 2 - each created by two promoter SNPs [rs3124952 (-986) and rs3124953 (-602) or rs7865453 (-64) and rs17514136 (-4), respectively], block 3 [involving three SNPs: exon 8 rs7851696 (+6424), rs73664188 and rs11103564, both 3’UTR] and block 4 [created by 3’UTR rs11103565, rs6537958, rs6537959 and presumably nine others variants] (**Figure 2**). As mentioned above, rs4521835 was excluded from analysis. Four haplotypes corresponding to block 3 were identified. Among them GTT (the reference genotype) and GTC had frequency >1%. Within the block 4, three haplotypes were found, including GTA (the reference genotype) and ATA with frequency >1%. The D’ between 3 and 4 blocks was 0.85. The frequency of haploblocks identified in extremely/early and moderate/late preterm neonates is presented in **Supplementary Materials** (**Figure S2**). In the case of neonates born before 33 week of gestation, data analysis gave five haploblocks, whereas for those having gestational age ≥33 weeks at birth, only three.

### The Diplotype-Phenotype Association

**Figure 3:**
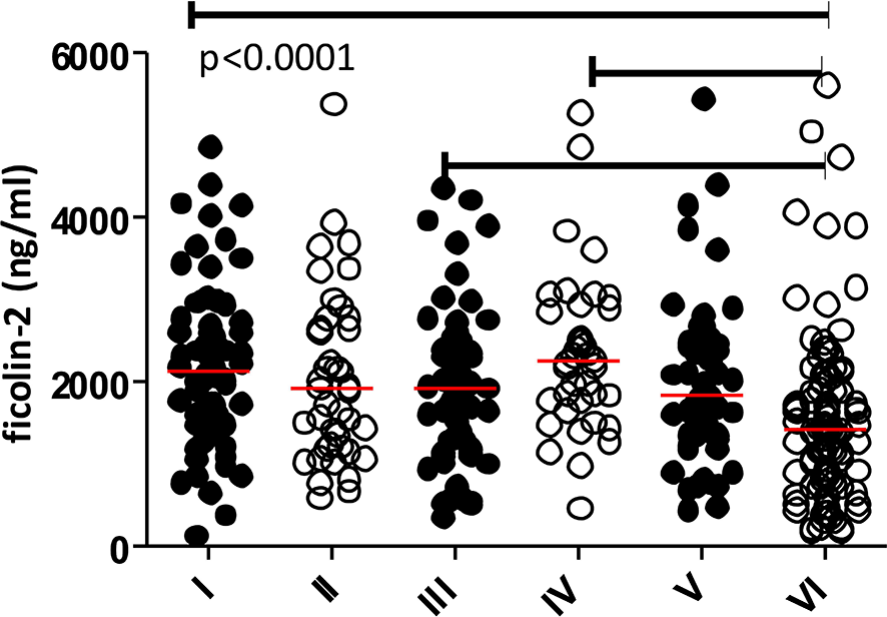
Individual concentrations of ficolin-2 in cord serum samples from preterm neonates in diplotype subgroups. Medians are shown as red bars. Median for the VIth subgroup differs significantly from those for the Ist, IIIrd and IVth ones.

PHASE software allowed us to identify 49 diplotypes (D1-D49), corresponding to block 3 and block 4 genetic variants: exon 8 rs7851696 (+6424, G>T) and five 3’UTR SNPs: rs4521835 (T>G), rs73664188 (T>C), rs11103564 (T>C), rs11103565 (G>A) and rs6537959 (T>A). Twenty of them, with frequency ≥1% are listed in the **Table 5**, whereas all identified diplotypes are presented in the **Supplementary Materials** (**Table S1**). GTTTGT/GTTTGT (D15) represents the reference diplotype. Diplotypes differing only in SNPs with no greater influence on ficolin-2 level, were grouped into five subgroups (I-V). The sixth subgroup was associated with relatively low ficolin-2 concentration (**Table 6** and **Figure 3**). There were no significant differences in gestational age or birthweight between subgroups. Two diplotypes: D28, (GTTTGT/GGTTGA) and D35 (GGTCGT/GGTCGA) with frequency of 1% were not included in any subgroup.

**Table 5:** The subgroups including identified diplotypes (with the frequency ≥1%*) in preterm neonates.

Subgroup	Diplotype			Ficolin-2 serum concentration (ng/ml)		
	Sequence	Description	Frequency (%, n)	n	Median	Range
I	GTTTGT/GTTTGT	D15	18.7 (94)	70	2154	153-4893
	GTTTGT/GTTCGT**	D6	1.4 (7)	4	2817	1637-3747
II	GTTTGT/GGTCGT	D13	6.7 (34)	28	2053	611-3957
	GTTTGT/GGTCGA	D10	3.6 (18)	14	1601	803-5408
III	GTTTGT/GGTCAA	D1	16 (80)	63	1932	372-4381
IV	GGTTGT/GGTCAA	D9	6.1 (31)	27	2285	481-5299
	GGTTGT/GGTCGA	D23	1.6 (8)	6	2277	1455-4891
	GGTTGT/GGTCGT	D22	1.2 (6)	5	2257	1166-3642
V	GGTCAA/GGTCAA	D3	5 (25)	19	1549	706-4165
	GGTCGT/GGTCAA	D4	4.1(21)	18	1923	520-3646
	GGTCGA/GGTCAA	D11	4 (20)	18	1785	479-5481
VI***	GTTTGT/TGCTGT	D2	5.7 (29)	22	1479	204-4081
	GTTTGT/GGCTGT	D17	4 (20)	16	1132	242-5644
	GGTCAA/TGCTGT	D14	3.6 (18)	16	1383	478-2157
	GGTCAA/GGCTGT	D5	2.6 (13)	12	1410	237-5068
	GGTTGT/TGCTGT	D7	1.8 (9)	9	1737	976-3935
	GGTCGT/TGCTGT	D32	1.4 (7)	4	1075	504-1456
	GGTCGT/GGCTGT	D19	1.2 (6)	3	2148	1934-2196
	GGTTGT/GGCTGT	D21	1.2 (6)	5	2323	1266-2657
	TGCTGT/TGCTGT	D12	1.0 (5)	5	937	331-2194

* - as mentioned, D28 and D35 were not included in any subgroup; ** - the differing nucleotide (in comparison with the most common diplotype within subgroup is underlined; *** - in the subgroup VI, all diplotypes with frequency of ≥1% with G allele at rs4521835 and C at rs73664188 (marked in red) are collected. For D12, the T allele at rs7851696 (+6424, G>T) related also with ficolin-2 low level is also marked.

**Table 6:** Associations of the *FCN2* gene 3’UTR diplotypes with adverse effects of prematurity.

	Diplotype group:						
	I	II	III	IV	V	VI	I-VI
	D6, D15	D10, D13	D1	D9, D22, D23	D3, D4, D11	D2, D5, D7, D14, D17, D19, D32	
**n**	102	51	80	46	66	122	467
**Median ficolin-2 concentration (ng/ml) (n)**	2178 (74)	1938 (41)	1914 (63)	2271 (38)	1831 (55)	1438 (100)	1831 (371)
**Median gestational age (weeks)**	34	35	35	34.5	35	35	35
**Median birthweight (g)**	2305	2520	2270	2430	2310	2305	2320
**Gestational age <33 weeks**	22/102 (21.6%)	14/51 (27.5%)	17/77 (22%)	5/44 (11.4%)	13/66 (19.7%)	25/122 (20.5%)	96/462 (20.8%)
	ns	ns	ns	ns	ns	ns	
**Birthweight ≤1500g**	12/102 (11.8%)	6/51 (11.8%)	10/77 (13%)	2/44 (4.5%)	1/66 (1.5%)	20/122 (16.4%)	51/462 (11%)
					p=0.005, OR=0.11,	p=0.042, OR=1.95,	
	ns	ns	ns	ns	95% CI (0.01-0.78)*	95% CI (1.07-3.58)*	
**Early onset of perinatal infection**	13/100 (13%)	14/49 (28.6%)	10/78 (12.8%)	5/43 (11.6%)	7/64 (10.9%)	17/117 (14.5%)	66/451 (14.6%)
		p=0,008, OR=2.69,					
	ns	95% CI (1.36-5.34)*	ns	ns	ns	ns	
**Late onset of perinatal infection**	8/100 (8%)	3/49 (6.1%)	6/78 (7.7%)	2/43 (4.7%)	3/64 (4.7%)	10/117 (8.5%)	32/451 (7.1%)
	ns	ns	ns	ns	ns	ns	
**Pneumonia**	12/102 (11.8%)	9/51 (17.6%)	4/79 (5.1%)	3/43 (7%)	5/65 (7.7%)	5/120 (4.2%)	38/460 (8.3%)
		p=0.026, OR=2.81,				ns; p=0.08, OR=0.4,	
	ns	95% CI (1.25-6.33)*	ns	ns	ns	95% CI (0.15-1.06)*	
**RDS**	15/101 (14.9%)	10/51 (19.6%)	18/79 (22.8%)	8/43 (18.6%)	16/66 (24.2%)	29/119 (24.4%)	96/459 (20.9%)
	ns; p=0.074, OR=0.57, 95% CI (0.31-1.04)*	ns	ns	ns	ns	ns	
**pPROM**	18/101 (17.8%)	5/50 (10%)	10/76 (13.2%)	10/44 (22.7%)	11/66 (16.7%)	19/119 (16%)	73/456 (16%)
	ns	ns	ns	ns	ns	ns	

* - OR and 95% CI are given when p<0.1; ns, not significant.

**Figure 4:**
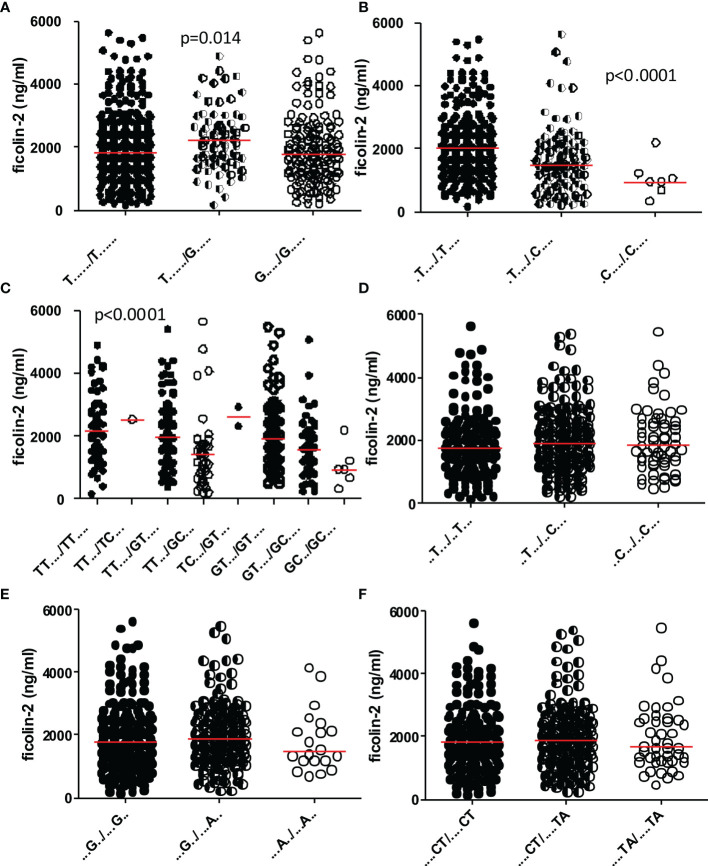
The influence of selected single nucleotide polymorphisms on ficolin-2 cord blood serum concentration in preterm neonates. Genetic variants: rs4521835 **(A)**, rs73664188 **(B)**, rs4521835 and rs73664188 **(C)**, rs11103564 **(D)**, rs11103565 **(E)**, rs6537958 and rs6537959 **(F)**. Medians are shown as red bars. Ficolin-2 concentrations among carriers of various genotypes were compared using Kruskal-Wallis ANOVA.

The diplotype association with ficolin-2 concentration in cord serum was found to be markedly influenced by SNPs at rs4521835 and rs73664188 (**Figures 4A, B**). Genetic variant GC (present in D2, D5, D7, D12, D14, D17, D19, D21 and D32 diplotypes, included in subgroup VI; **Table 5**) was associated with low ficolin-2 serum concentration. The lowest median was associated with D12, where both haplotypes carry GC variants. Additionally, D12 represents the T allele at rs7851696 (+6424), also associated with low ficolin-2 level in cord serum (data not shown). The trend towards lower protein concentrations was found for the A variant of rs11103565 as well as TA variants at rs6537958 and rs6537959, being in perfect LD (**Figure 4F**). No association was observed for rs11103564 (**Figure 4D**). Interestingly, T/G heterozygosity at rs4521835 seems to be related to a higher protein concentration (**Figure 4A**).

Besides the relationship of 3’UTR diplotypes with ficolin-2 cord serum concentration described above, the association of genotype with selected complications like early prematurity, low birthweight, susceptibility to perinatal infection, pPROM and RDS was analyzed (**Table 6**).

It revealed that the probability of carrying one of subgroup II diplotypes, [heterozygous at rs4521835: GTTTGT/GGTCGT (D13) and GTTTGT/GGTCGA (D10)] is significantly higher among neonates with early onset of infection or pneumonia (OR=2.69, p=0.008 and OR=2.81, p=0.026, respectively) than in the group of neonates without such complications (**Table 6**). Diplotypes representing subgroup V (homozygous for C allele at rs11103564) were less frequent among babies born with birthweight ≤1500 g (OR=0.11 p=0.005) than among neonates born with a higher body mass. In contrast the over-representation of subgroup VI diplotypes with GC variants at rs4521835 and rs73664188 was found among newborns with the same birthweight range (OR=1.95, p=0.042). Moreover, that association seems to be independent of multiple pregnancies, since after excluding twins and triplets, the OR was even higher (2.98, p=0.003). No association of diplotypes with other prematurity-related complications was found.

### *In Silico* Functional Analysis

SNPinfo (FuncPred) software was used for *in silico* determination of the interactions of genetic variants with microRNA (**Table 7**). Only rs4521835 was predicted to be a target for microRNA (miR-150, miR-484, miR-618 miR-1226 and miR-1229). The regulomeDB database [designed to assess the probability of particular variant that affects binding of transcription factor (35)] helped to determine the regulatory function of SNPs investigated, with lower score indication of more evidence of functionality (36). Rs11103564 with score of 1f was predicted to be linked to expression of a gene target and to be an eQTL (expression quantitative trait locus) for *OLFM1* (olfactomedin 1), whereas rs4521835, rs7847431 and rs11462298 with rank of 3a are less likely to be associated within the functional region. The remaining genetic variants with score 4-5 have minimal evidence of binding. Variants of rs4521835, rs11103565, rs7040372, rs7046516, rs7847431 and rs11462298 may alter significantly transcription factor (TF) binding motifs.

**Table 7:** *In silico* prediction of functional role of analyzed genetic variants.

	SNPinfo	RegulomeDB		
	miRNA binding	Score	Binding motif for TF	eQTL
**rs4521835**	**+**	3a	RREB1 TERF1	-
**rs73664188**	–	4	–	–
**rs11103564**	–	1f	–	OLFM1
**rs11103565**	–	5	ELF4	–
**rs7040372**	–	5	ZSCAN26	–
**rs7046516**	–	5	HNF4A	–
**rs7847422**	–	4	–	–
**rs7847431**	–	3a	EWSR1	
**rs6537957**	–	4	–	–
**rs6537958**	–	4	–	–
**rs6537959**	–	4	–	–
**rs6537960**	–	4	–	–
**rs6537962**	–	4	–	–
**rs11462298**	–	3a	RREB1	–
			TERF1	
**rs7860507**	–	4	–	-

### The Impact of the *FCN2* Gene 3’UTR Genetic Variants on Protein Binding

**Figure 5:**
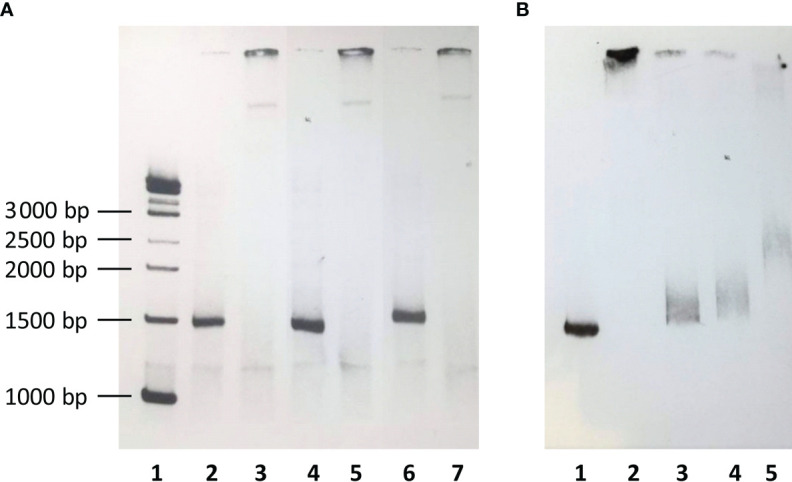
**(A)** The electrophoretic mobility of biotin-labelled DNA fragment including polymorphic sites at rs11103565, rs7040372, rs7046516, rs7847422, rs7847431, rs6537957, rs6537958, rs6537959, rs6537960, rs6537962, rs11462298 and rs7860507. 1 – biotinylated molecular weight marker; 2, 3 - major allele homozygote; 4, 5 - variant allele homozygote; 6, 7 – heterozygote; lanes 2, 4, 6 – with no nuclear extract; lanes 3, 5, 7 – preincubated with nuclear extract. **(B)** Inhibition of binding of nuclear extract to aforementioned biotin-labelled DNA fragment from major allele homozygote. 1 – no nuclear extract, no inhibitor; 2 – nuclear extract, no inhibitor; 3 – nuclear extract, unlabelled DNA from major alelle homozygote as inhibitor; 4 - nuclear extract, unlabelled DNA from minor alelle homozygote as inhibitor; 5 - nuclear extract, unlabelled, unrelated DNA as inhibitor (1122 bp PCR product, corresponding to the 134 887 339-134 888 460 region of *FCN2* gene.

Sanger sequencing and LD analysis revealed a perfect linkage among eleven SNPs (rs7040372, rs7046516, rs7847422, rs7847431, rs6537957, rs6537958, rs6537959, rs6537960, rs6537962, rs11462298 and rs7860507) and a very high LD with rs11103565. In order to investigate the biological significance of non-random perfect linkage of twelve SNPs, their impact on nuclear protein binding was tested. Biotin-labelled 1325 bp PCR products of DNA samples from homozygotes for reference/variant alleles as well as from heterozygotes were incubated with nuclear extract. A significant shift in electrophoretic mobility was observed in all cases (**Figure 5A**). Moreover, the interaction between biotinylated wild-type DNA and nuclear extract proteins was inhibited not only by corresponding unlabelled PCR product but also by DNA of homozygote for variant alleles as well as by unrelated DNA PCR product (**Figure 5B**).

## Discussion

Ficolin-2 has been associated with a variety of disorders (3, 32, 34, 37–42) and proposed as a potential biomarker of certain clinical conditions. Previous studies have demonstrated that ficolin-2 serum concentration/activity is significantly affected by polymorphisms located in both the promoter region and exon 8 (15, 16, 18). However, marked intra-genotype variations in ficolin-2 levels can complicate disease association studies.

The 3’-untranslated region may be involved in regulation of gene expression by controlling mRNA nuclear export, cytoplasmic localization and stability. It may be a target for transcriptional factors and microRNAs. 3’UTR polymorphic variants may create or abolish their binding sites, or modulate enhancer/silencer effects. The influence of 3’UTR variants on concentration of corresponding proteins in the circulation has been demonstrated previously. For example, Ning and Zhang (43) described the impact of two *PTX3* gene 3’UTR SNPs on pentraxin-3 level in the Chinese Han population. Doi et al. (44) described the association of two *VEGF* gene 3’UTR polymorphisms with plasma VEGF levels in Japanese. Furthermore, Zanetti et al. (28) and Świerzko et al. (29) found *MBL2* gene 3’UTR polymorphisms to influence mannose-binding lectin (MBL) serum concentration. The polymorphisms in the *FCN2* gene 3’UTR were still unexplored and to our knowledge, this is the first report to investigate this issue in depth. Previously, there was only one report describing a trend towards a higher frequency of the G allele at rs4521835 in Polish kidney allograft recipients with delayed graft function (45).

We report here 15 genetic variants with MAF >1% in the *FCN2* 3’UTR in a cohort of 504 Polish preterm neonates. Their frequencies were similar to the frequencies reported for Caucasian/Central European populations (**Table 4**). RegulomeDB predicted four SNPs (rs11103564, rs4521835, rs7847431and rs11462298) to lie in the functional location and likely to result in functional consequences (**Table 7**).

Eleven SNPs (rs7040372, rs7046516, rs747422, rs7847431, rs6537957, rs6537958, s6537959, rs6537960, rs6537962, rs11462298 and rs7860507) were shown to be in complete linkage disequilibrium and together with rs73664188, rs11103564 and rs11103565 as well as with exon 8 rs7851696 (+6424) formed two related haplotype blocks. Among 49 diplotypes created from rs7851696 (G>T), rs4521835 (T>G), rs73664188 (T>C), rs11103564 (T>C), rs11103565 (G>A) and rs6537959 (T>A), twenty two occurred with frequency ≥1% (**Table 5**). Rs4521835 - due to deviation from HWE - was excluded from haplotype creation by Haploview software, but it was included in diplotypes reconstructed by PHASE software. Interestingly, deviation from HWE for rs4521835 was observed in neonates with gestational age ≥33 weeks only, but not in the neonates born before 33rd week of pregnancy.

Two polymorphisms (rs4521835 and rs73664188) were shown to significantly influence ficolin-2 concentration in cord serum, with the lowest levels observed in the GC haplotype carriers (**Table 5** and **Figures 3**, **4**). When the diplotype-serum concentration relationship was analyzed, the lowest ficolin-2 levels were shown in individuals carrying the C allele at rs73664188, the G allele at rs4521835 and the T allele at exon 8 rs7851696 (+6424) at least on one strand (**Table 5**). Interestingly, the SNPinfo database indicated possible involvement of rs4521835 genetic variants in creating or abolishing miRNA targets sites, which may suggest epigenetic regulation of ficolin-2 synthesis. Moreover, the rs73664188 SNP seems to modify an impact of promoter polymorphisms on ficolin-2 concentration in cord serum (**Figure S3**).

There have been some reports of significant differences in *FCN2* genotypes (involving promoter and/or structural regions) between patients and healthy controls in several clinical contexts (34, 39, 46–49). The results presented here demonstrate an association of subgroup II diplotypes D13 (GTTTGT/GGTCGT) and D10 (GTTTGT/GGTCGA) among preterm neonates with early onset of infection and/or pneumonia (OR in range 2.69-2.81, p<0.05, in comparison with newborns with no infections till hospital discharge). Moreover, in the group of neonates with birthweight ≤1500 g the lower frequency of subgroup V genotypes (both C allele at rs11103564, OR=0.11, p=0.005) but higher probability of subgroup VI diplotypes [associated with low ficolin-2 level resulting from GC variants at rs4521835 and rs73664188 (OR=1.95, p=0.042)] were observed. The assumption that carrying C allele at rs73664188 is associated with low birthweight is supported by an increased odds ratio when only data from singleton pregnancies were analyzed (OR=2.98, p=0.003). Therefore, this variant seems to be a risk factor for one of the adverse effects of prematurity.

Eleven SNPs stretched on the distance of 1242 bp analyzed here were shown to be in perfect LD and with very high LD with rs11103565. They had no greater impact on ficolin-2 concentration in cord sera, however some of them were predicted by RegulomeDB to be associated with transcription factor binding (rs7040372, rs7046516, rs7847431 and rs11462298). We assumed that absolute LD between eleven SNP may be associated with nuclear protein binding. Polymorphism of DNA and RNA 3’ untranslated regions may modulate such interactions. For example, the 3’UTR T variant of the *XRCC2* gene at rs3218550 was shown to enhance nuclear protein binding (50). Using a gel shift assay, we tested whether 1325 bp PCR product including twelve SNPs may be involved in protein binding and whether such interaction may be influenced by their genetic variants. Our results indicate that three tested genetic variants were able to bind nuclear extract factors. The results are not easy to interpret since the protein-DNA interaction can be either DNA sequence-nonspecific (protein-sugar phosphate DNA backbone interactions) or DNA sequence-specific (protein-base pair interactions) (51). The inhibition of nuclear extract induced electrophoretic mobility of biotinylated wild type DNA with variant alleles homozygote DNA may suggest non-allele specific binding, however partial inhibition by an unrelated DNA PCR product may indicate partially specific interaction between wild type DNA and nuclear protein(s).

In addition to RegulomeDB, *in silico* analysis using PROMO software, 31 different transcription factors (TF) were predicted to be able to recognise putative binding sites including 12 SNPs. Five of them were able to bind to major allele variants only, and seven to minor variants only, whereas fourteen were predicted to be reactive independently of genotype. For example p53 was shown to possibly interact with both variants at rs7046516, rs7847422 and rs6537957. In contrast to other TFs, p53 DNA targets are not defined by a particular consensus sequence, but it is able to bind various DNA sequences and DNA targets defined by their secondary structures and the majority of p53 target structures are out with the promoter region [reviewed by Brazda and Fojta (52)]. Moreover, p53 was shown to interact with other TFs, like YY-1, which was predicted to interact at rs7040372, rs7847422 and rs6537962. This, among other things, makes the elucidation of the molecular basis of the interactions of the nuclear extract factors with the *FCN2* 3’UTR region more difficult.

To summarize, sequencing of the 3’UTR of the *FCN2* gene in a relatively large cohort of preterm babies and a multiway *in silico* analysis allowed us to find potential associations of certain polymorphic variants of that region with common adverse effects of prematurity. Furthermore, some polymorphisms were demonstrated to affect ficolin-2 concentration in cord serum, although the associations mentioned may reflect linkage disequilibrium with promoter/coding region SNP. The 3’UTR polymorphism can be also involved in nuclear protein binding.

## Data Availability Statement

The raw data supporting the conclusions of this article will be made available by the authors, without undue reservation.

## Ethics Statement

The studies involving human participants were reviewed and approved by Bioethics Committee of The Karol Marcinkowski Poznań University of Medical Sciences; Independent Bioethics Committee for Scientific Research at Medical University of Gdańsk; Bioethics Committee of The Medical University of Łódź. Written informed consent to participate in this study was provided by the participants’ legal guardian/next of kin.

## Author Contributions 

AŚ coordinated project realisation, designed the study, planned and supervised experimental work, determined ficolin-2 concentrations, sequenced *FCN2* gene 3’UTR and determined SNP at -986/-602, analyzed experimental data. DJ and MMi designed the procedure for *FCN2* gene 3’UTR sequencing and produced corresponding data. DJ determined SNP using TaqMan probes; analyzed haplotypes and preformed EMSA experiments. GG determined SNP at -64/-4/+6359/+6424 and performed EMSA experiments. AS-P analyzed diplotypes. KC, PK, MK-B, and KS were responsible for patients’ qualification, taking and collecting samples as well as collecting clinical data. JM, ID-P, and JK supervised qualification and recruitment of patients and analysis of clinical data. MMa and HS provided anti-ficolin-2 mAbs for initial experiments, contributed to modification of the procedure and discussed corresponding data. DK contributed to the study design and data analysis. MC contributed to the study design, supervision of experimental work, analyzed haplotypes and performed statistical analysis. Draft manuscript was written by AŚ, DJ, and MC, and revised by DK and MC. All authors contributed to the article and approved the submitted version.

## Funding

This work was funded by National Science Centre, Poland, grant 2015/17/B/NZ6/04250.

## Conflict of Interest

The authors declare that the research was conducted in the absence of any commercial or financial relationships that could be construed as a potential conflict of interest.

## Publisher’s Note

All claims expressed in this article are solely those of the authors and do not necessarily represent those of their affiliated organizations, or those of the publisher, the editors and the reviewers. Any product that may be evaluated in this article, or claim that may be made by its manufacturer, is not guaranteed or endorsed by the publisher.
