# Supplementary material for: Polymorphisms of the FCN2 Gene 3’UTR Region and Their Clinical Associations in Preterm Newborns
Source: Front Immunol. 2021 Oct 28;12:741140. doi: 10.3389/fimmu.2021.741140 (PMC8581395; doi:10.3389/fimmu.2021.741140)
Supplement: Supplementary file 1 [file Image_1.pdf]

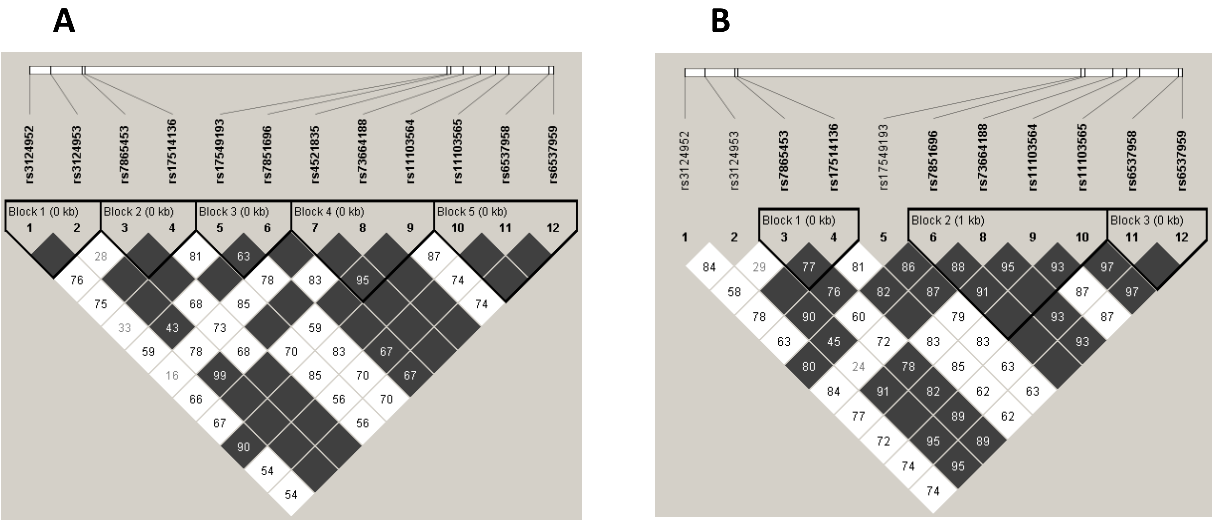

Figure S1. Linkage disequilibrium analysis of promoter [rs3124952 (-986); rs3124953 (-602); rs7865453 (-64); rs17514136 (-4)], exon 8 [rs17549193 (+6359); rs7851696 (+6424)] SNP and polymorphisms identified in this study (rs4521835, rs73664188; rs11103564; rs11103565; rs6537958; rs6537959) in extremely/early (**A**) and moderate/late (**B**) preterm neonates. The numbers in the grid refer to D’ parameter (presented as percent) of the given pairs of SNPs. Empty grey squares represent perfect LD (D’=1). SNP identifiers are indicated on the abscissas. Bolded triangles shows haplotype blocks identified using Four Gamete Rule test.
