# Supplementary material for: Polymorphisms of the FCN2 Gene 3’UTR Region and Their Clinical Associations in Preterm Newborns
Source: Front Immunol. 2021 Oct 28;12:741140. doi: 10.3389/fimmu.2021.741140 (PMC8581395; doi:10.3389/fimmu.2021.741140)
Supplement: Supplementary file 2 [file Image_2.pdf]

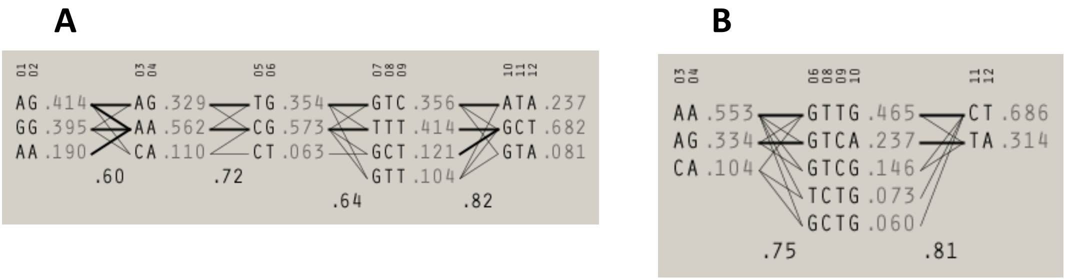

Figure S2. The haplotype blocks identified in extremely/early (**A**) and moderate/late (**B**) preterm neonates using Four Gamete Rule test. The frequency and the level of recombination between blocks are given. 01 - rs3124952 (-986); 02 - rs3124953 (-602); 03 - rs7865453 (-64); 04 - rs17514136 (-4), 06 - rs7851696 (+6424), 08 - rs73664188; 09 - rs11103564; 10 - rs11103565; 11 - rs6537958; 12 - rs6537959. A multiallelic D' statistic, which indicates the level of recombination between two blocks, is shown in the crossing area.
