# Supplementary material for: Polymorphisms of the FCN2 Gene 3’UTR Region and Their Clinical Associations in Preterm Newborns
Source: Front Immunol. 2021 Oct 28;12:741140. doi: 10.3389/fimmu.2021.741140 (PMC8581395; doi:10.3389/fimmu.2021.741140)
Supplement: Supplementary file 3 [file Image_3.pdf]

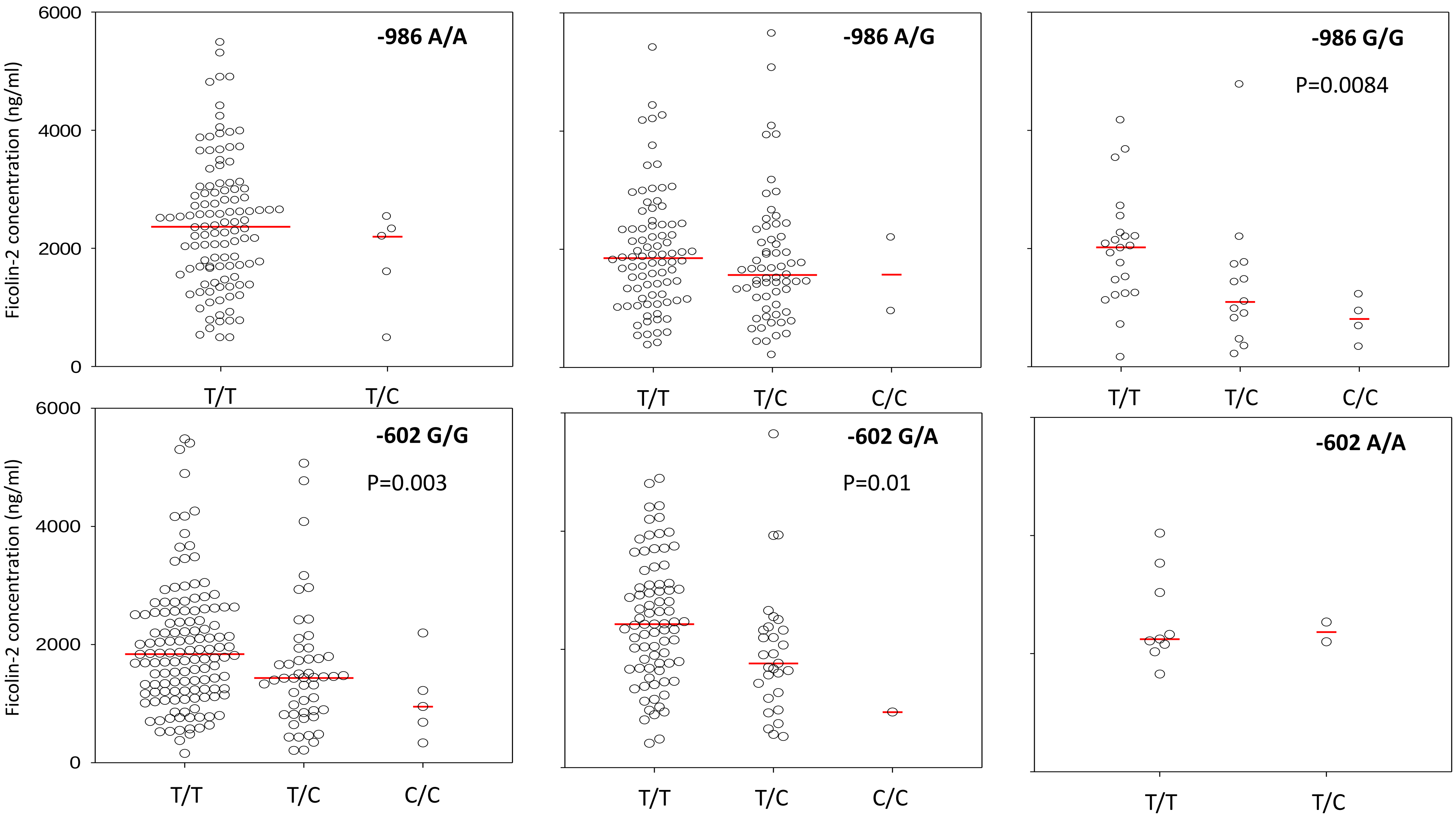
Ficolin-2 concentration (ng/ml)
6000
4000
2000
0
-986 A/A
T/T
T/C
-986 A/G
T/T
T/C
C/C
-986 G/G
P=0.0084
T/T
T/C
C/C
Ficolin-2 concentration (ng/ml)
6000
4000
2000
0
-602 G/G
P=0.003
T/T
T/C
C/C
-602 G/A
P=0.01
T/T
T/C
C/C
-602 A/A
T/T
T/C

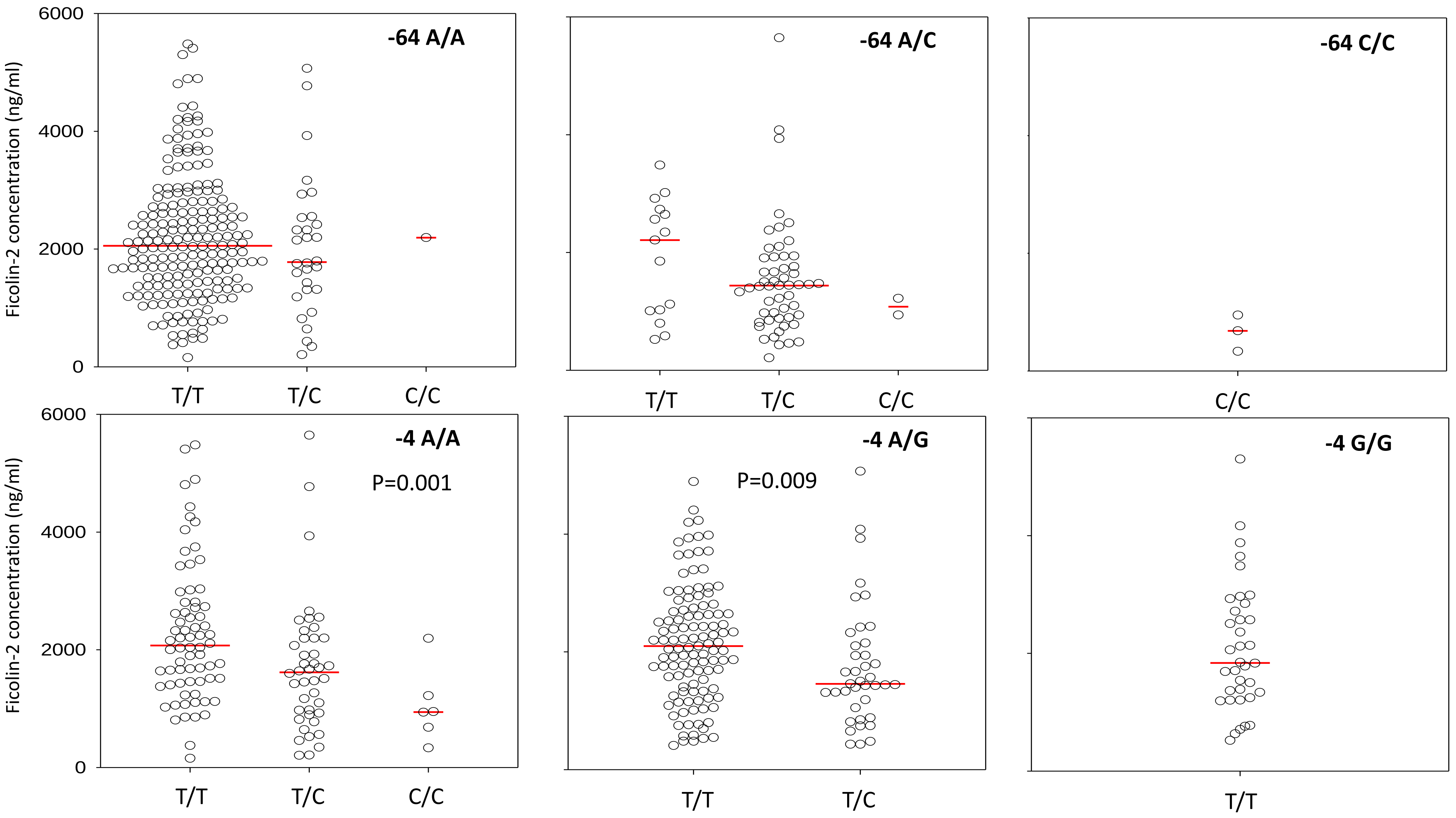

Figure S3. Influence of the *FCN2* gene 3'UTR rs73664188 polymorphism on promoter SNP-dependent concentration of ficolin-2 in cord sera from moderate/late preterm neonates. Data related to rs73664188 T/T, T/C and C/C variants were compared in association with major allele homozygosity, heterozygosity and minor allele homozygosity at positions -986, -602, -64 and -4. Data were compared using Kruskal-Wallis ANOVA test. Statistical significance is shown when p<0,05 only.
