# Supplementary material for: Polymorphisms of the FCN2 Gene 3’UTR Region and Their Clinical Associations in Preterm Newborns
Source: Front Immunol. 2021 Oct 28;12:741140. doi: 10.3389/fimmu.2021.741140 (PMC8581395; doi:10.3389/fimmu.2021.741140)
Supplement: Supplementary file 4 [file Table_1.docx]

**Table S1**. Diplotypes identified in this study – their frequencies and association with ficolin-2 concentration in serum.

| **Diplotype** | | **Frequency % (n)** | **Ficolin-2 serum concentration (ng/ml)** | | |
| --- | --- | --- | --- | --- | --- |
|  |  |  | **n** | **median** | **range** |
| **D1** | GTTTGT/GGTCAA | 15.9% (80) | 62 | 1932 | 372-4381 |
| **D2** | GTTTGT/TGCTGT | 5.75% (29) | 23 | 1471 | 204-4081 |
| **D3** | GGTCAA/GGTCAA | 5% (25) | 19 | 1549 | 706-4165 |
| **D4** | GGTCGT/GGTCAA | 4% (21) | 18 | 1923 | 520-3646 |
| **D5** | GGTCAA/GGTCAA | 2.6% (13) | 12 | 1410 | 237-5068 |
| **D6** | GTTTGT/ GTTCGT | 1.4% (7) | 4 | 2817 | 1637-3747 |
| **D7** | GGTTGT/TGCTGT | 1.8% (9) | 9 | 1737 | 976-3935 |
| **D8** | GGTCAA/GGCTGA | 0.2% (1) | 1 | 3167 | - |
| **D9** | GGTTGT/GGTCAA | 6% (31) | 27 | 2285 | 481-5299 |
| **D10** | GTTTGT/GGTCGA | 3.6% (18) | 14 | 1601 | 803-5408 |
| **D11** | GGTCGA/GGTCAA | 4% (20) | 18 | 1785 | 479-5481 |
| **D12** | TGCTGT/TGCTGT | 1% (5) | 5 | 937 | 331-2194 |
| **D13** | GTTTGT/GGTCGT | 6.7% (34) | 27 | 2053 | 611-3957 |
| **D14** | GGTCAA/TGCTGT | 3.6% (18) | 16 | 1383 | 478-2157 |
| **D15** | GTTTGT/GTTTGT | 18.7% (94) | 70 | 2145 | 153-4893 |
| **D16** | GGTCGA/GGCTGT | 0.6% (3) | 3 | 2417 | 430-2504 |
| **D17** | GTTTGT/GGCTGT | 4% (20) | 16 | 1132 | 242-5644 |
| **D18** | GGTCGA/GGTCGA | 0.2% (1) | 1 | 1249 |  |
| **D19** | GGTCGT/GGCTGT | 1.2% (6) | 3 | 2148 | 1934-2196 |
| **D20** | GGTTGA/GGTCAA | 0.4% (2) | 2 | 1352 | 1256-1477 |
| **D21** | GGTTGT/GGCTGT | 1.2% (6) | 5 | 2323 | 1266-2657 |
| **D22** | GGTTGT/GGTCGT | 1.2% (6) | 5 | 2257 | 1166-3642 |
| **D23** | GGTTGT/GGTCGA | 1.6% (8) | 6 | 2277 | 1455-4891 |
| **D24** | GGTCGT/GGTCAT | 0.2% (1) | 1 | 2298 | - |
| **D25** | GTCTGT/GGTCAA | 0.2% (1) | 1 | 2324 | - |
| **D26** | GTTTGT/GTCTGT | 0.4% (2) | 1 | 2534 | - |
| **D27** | GTTTGT/TGCTGT | 0.2% (1) | 1 | 923 | - |
| **D28** | GTTTGT/GGTTGA | 1% (5) | 1 | 4805 | - |
| **D29** | GGTCGA/TGCTGT | 0.6% (3) | 3 | 1097 | 424-2439 |
| **D30** | GGTTGT/GGTTGA | 0.4% (2) | 2 | 1262 | 1067-1457 |
| **D31** | GGTTGA/GGCTGT | 0.2% (1) | - | - | - |
| **D32** | GGTCGT/TGCTGT | 1.4% (7) | 4 | 1075 | 504-1456 |
| **D33** | GTCTGA/GGTCAA | 0.2% (1) | 1 | 2931 | - |
| **D34** | GTTTGT/TGTTGT | 0.2% (1) | - | - | - |
| **D35** | GGTCGT/GGTCGA | 1% (5) | 1 | 765 | - |
| **D36** | GTTTGT/GGTCAA | 0.2% (1) | 1 | 1167 | - |
| **D37** | GGTCGT/TGTCAA | 0.2% (1) | 1 | 3483 | - |
| **D38** | GTTTGT/TTTTGT | 0.2% (1) | 1 | 4259 | - |
| **D39** | GGTCAT/GGTCAA | 0.2% (1) | - | - | - |
| **D40** | GGTCGA/TGTCAA | 0.2% (1) | 1 | 2997 | - |
| **D41** | GTTTGT/GGTTAA | 0.4% (2) | 2 | 3527 | 2646-4400 |
| **D42** | GGCTGT/GGCTGT | 0.4% (2) | 1 | 947 | - |
| **D43** | GTTTGT/GGCTGA | 0.2% (1) | 1 | 557 | - |
| **D44** | GGTTGT/GTTTGT | 0.2% (1) | 1 | 2188 | - |
| **D45** | GTTTGT/GGTCAT | 0.2% (1) | 1 | 1387 | - |
| **D46** | GGTCAA/TGTTGT | 0.2% (1) | 1 | 794 | - |
| **D47** | GGTTAA/GGTCAA | 0.2% (1) | 1 | 1192 | - |
| **D48** | GGTCGT/GGTCGT | 0.4% (2) | - | - | - |
| **D49** | GGTCGT/GGCCAA | 0.2% (1) | 1 | 2100 | - |
